# Analysis of microsatellite instability in colorectal carcinoma by microfluidic-based chip electrophoresis

**DOI:** 10.1136/jcp.2008.056994

**Published:** 2009-08-20

**Authors:** M Odenthal, N Barta, D Lohfink, U Drebber, F Schulze, H P Dienes, S E Baldus

**Affiliations:** 1 Institute of Pathology, University Hospital of Cologne, Germany; 2 Institute of Pathology, University of Duesseldorf, Germany

## Abstract

Microsatellite analysis is an important tool in clinical research and molecular diagnostics because microsatellite instability (MSI) occurs frequently in various types of cancer. Approximately 10–15% of colorectal, gastric and endometrial carcinomas are associated with MSI, and this has an impact on clinical prognosis. The microsatellite loci Bat25, Bat26, D2S123, D5S346 and D17S250, recommended by the Bethesda guidelines, were analysed by microfluidic-based on-chip electrophoresis in 40 cases of colon carcinoma with known MSI status. In all cases, microfluidic separation of the PCR amplicons resulted in highly resolved, distinct patterns of each of the five microsatellite loci. Detection of MSI could be demonstrated by microsatellite-loci-associated, well-defined deviations in the electropherogram profiles of tumour and non-tumour material, and confirmed the classification of MSI cases performed by conventional technology. In conclusion, microfluidic chip technology is a simple and reliable approach for MSI detection that allows label-free and very fast analysis of microsatellite amplicons.

In 10–20% of patients with colorectal cancer (CRC), carcinogenesis is due to genomic defects in the mismatch repair machinery. Defective DNA repair as a result of germ-line mutations has been linked to sporadic colorectal carcinoma, and also to those carcinomas arising in hereditary non-polyposis colorectal carcinoma (HNPCC) syndrome. In both settings, the mutations and promoter hypomethylation occur mainly in the genes h*MLH1* and h*MSH2* of the mismatch repair system, and result in loss of their expression.^1^ Further, defects in the mismatch repair process with subsequent base pair mismatches lead to microsatellite instability (MSI).^1^ ^2^ Since the failure of the repair system as a cause of genomic instability is associated with a better prognosis^1^ ^3^ many different microsatellite loci have been used to identify MSI in tumours for diagnostic and prognostic purposes.^2^ In an attempt to provide uniformity in clinical diagnoses, an international meeting at the National Cancer Institute (NCI) recommended primary microsatellite markers for use in CRC MSI testing in clinical and research settings. The recommended Bethesda MSI testing set comprises the microsatellite loci Bat25, Bat26, D2S123, D5S346 and D17S250, characterised by mononucleotide and dinucleotide repeats.^4^

Different technologies have demonstrated their applicability for MSI detection in the past, and fluorochrome-based PCR assays linked to capillary electrophoresis using a sequencing platform are most commonly used.^5^ We have focused on the analysis of microsatellite loci using microfluidic-based on-chip electrophoresis, because recent studies have shown microfluidics technology to be an electrophoresis method with high-resolution capacity combined with a short running time; these studies have been reviewed.^6^ In the present study, we investigate microfluidic-based chip devices as an analytical platform for MSI detection at all five loci known as the Bethesda panel.

## Materials and methods

### Tissue material

Human CRC tissues were collected at the Institute of Pathology, University Hospital of Cologne, Germany, in accordance with local research ethical guidelines. The tissues were fixed in formalin and embedded in paraffin as per routine protocol. From a panel of 150 colorectal carcinomas diagnosed according to the Bethesda guidelines,^4^ a total of 40 cases (14 MSI positive and 26 MSI negative) were chosen to analyse the suitability of on-chip electrophoresis for MSI diagnosis.

Two haematoxylin-stained sections (3 μm thick) were evaluated by two pathologists, and tumour as well as non-tumour areas were macrodissected manually for further analyses.

### DNA extraction and PCR

The macrodissected specimens were first deparaffinised and then lysed overnight at 56°C by proteinase K digestion (500 μg/ml proteinase K, 5 mM EDTA, 20 mM Tris pH 8.0). The DNA was extracted using a DNA-extraction kit from Qiagen (Hilden, Germany) according to the manufacturer’s instructions. The DNA yield was between 10 and 30 ng/μl, determined by A_260_ measurement using a Nanodrop spectrophotometer (PeqLab Biotechnologie, Erlangen, Germany). DNA extracts (2 μl) were applied in the Multiplex-PCR approach of Qiagen according to the manufacturer’s instructions, using 60°C in the annealing step. The details of the primer sets used for amplification of the microsatellite loci Bat25, Bat26, D2S123, APC-D5S346 and MFd15-D17S250 are shown in the supplementary table S1.^2^ ^5^ Primer sets of the Bat25 and the D2S123 loci, and of the D5S346 and the D17S250 loci, were combined in duplex assays.

### MSI analyses by on-chip electrophoresis

For the separation of microsatellite PCR products, we used DNA 1000 LabChip kits, which are manufactured for research purposes only. In brief, the chips were prepared with gel-dye mix, pressurised, and then marker solution and DNA 1000 ladder were added. For this process, 1 μl of each PCR reaction was pipetted into one out of 12 sample wells of a prepared chip. After vortexing, the chip was placed in the Agilent 2100 bioanalyser. The electrophoresis of 12 samples lasted approximately 30–40 min. Fragment analysis was carried out using the Agilent 2100 expert software, and an overlay of two electropherograms was used to compare PCR patterns derived from tumour and non-tumour tissues. Differences in the peak patterns of the overlaid electropherograms were evaluated. Three overlays per patient were used in order to identify instabilities in the microsatellite loci Bat25 and D2S123, D5S346 and D17S250, and Bat26.

In order to compare the MSI analysis by the lab-on-a-chip technology with a conventional method, loci were amplified with fluorochrome-labelled primer sets and analysed on a sequencing platform.^2^ ^5^

## Results and discussion

### Analysis of amplified microsatellite loci by microfluidic-based on-chip electrophoresis

**Figure 1:**
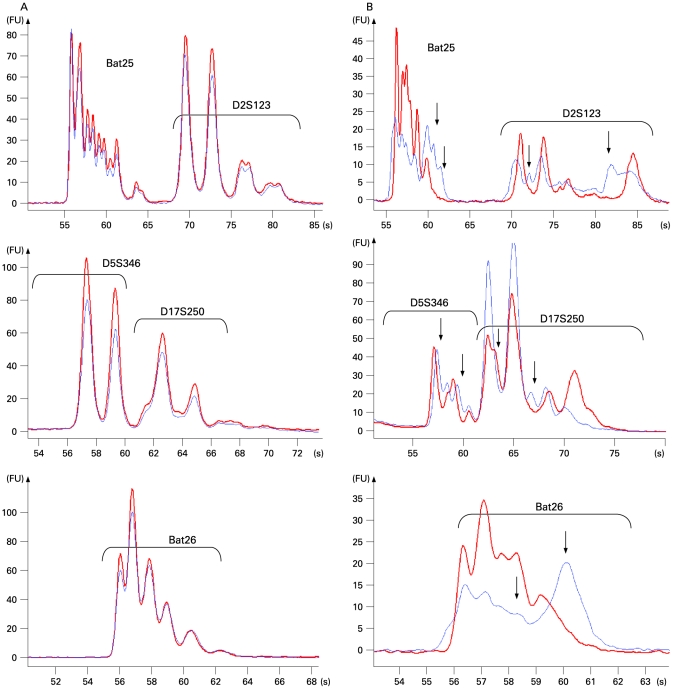
Overlay of electropherograms to classify the status of the five microsatellite loci. Electropherograms of microfluidic-based separation of unlabelled PCR products representing each of the five microsatellite loci Bat25+D2S123, D5S346+D17S250 and Bat26 are shown. Bat25 and Bat26 are mononucleotide repeats and D2S123, D5S346 and D17S250 are dinucleotide repeats. (A) The patterns of the electropherograms representing PCR amplification products derived from normal tissue (red) and tumourous tissue (blue) are perfectly matching and demonstrate microsatellite stability. However, the electropherogram overlays in (B) show significant deviations in the electrophoretic patterns of the microsatellite loci indicating microsatellite instability (arrows indicate divergent pattern of peaks).

The microsatellite loci of the Bethesda panel, Bat25, Bat26, D2S123, D5S346 and D17S250, recommended for CRC analyses by the conference at NCI,^4^ were amplified by label-free PCR. All five amplified microsatellite loci, including mononucleotide and dinucleotide repeats, were well resolved by microfluidic-based on-chip electrophoresis (fig 1). Previous work of Banerjea *et al* has shown that amplicons of the microsatellite locus Bat26 can be analysed by microfluidic-based electrophoresis and that this technology provides the advantage of being easy to use and highly standardised because of microfabrication of the chips.^7^ In our study, the analysis of all five recommended microsatellite loci took 15 min of preparation and no more than 30 min of electrophoretic resolving time in comparison with conventional procedures, which typically take between 4 and 6 h (see supplementary table S2). The benefits of microfluidic-based electrophoresis concerning low time and equipment operating expenses have also been shown in other molecular diagnostic approaches such as t(4,14) translocation,^8^ mutation analyses and quantification of promoter methylation.^9^

In addition to previous studies, we demonstrate herein that microfluidic-based analyses identify discrepancies in the microsatellite patterns between tumour and non-tumour DNA by the overlay of electropherograms (fig 1). In 25 cases, the electropherogram pattern of all five microsatellite amplicons from the tumour DNA exactly corresponded to the pattern obtained from non-tumour DNA (fig 1A), declaring the microsatellite status of these cases as stable (MSS). On the other hand, mismatches observed in the pattern of amplicons derived from tumourous and non-tumourous tissues clearly indicate microsatellite instability (fig 1B). Therefore, the microfluidic-based resolution appears to be an adequate technique to differentiate stable and instable microsatellite loci.

### Comparison of MSI analyses by microfluidic-based electrophoresis with conventional methods

Taken together, the assessment of the MSI status by microfluidic-based analysis shows more than 90% concordance with the results of conventional fluorescence-based, laser-associated detection methods. The MSI status of only two cases disagreed, with the MSI analyses by on-chip microfluidic-technology detecting an additional locus of instability in each case (table 1). In such cases, additional clinical information or data concerning the immunostaining of the repair enzymes hMLH1 and hMSH2 are helpful in deciding how to proceed further.^10^ In one discrepant case, negative immunostaining of the MLH1 repair enzyme pointed out that the MSI status proposed by the microfluidic-based electrophoresis appeared to be the correct interpretation.

**Table 1:** MSI status of colorectal carcinomas based on the results obtained by microfluidic-based chip analyses in comparison to prediagnosis

Samples, n (%)	Prediagnosis by fluorescence-linked PCR-approach and laser detection	Estimation by label-free PCR and microfluidics
12 (30)	MSI-H	MSI-H
1* (2.5)	MSI-L	MSI-H
1 (2.5)	MSI-L	MSI-L
1* (2.5)	MSS	MSI-L
25 (62.5)	MSS	MSS

MSI, microsatellite instability; MSI-H, instability in more than one microsatellite locus (*discrepant data between the techniques applied); MSI-L, instability in one microsatellite locus; MSS, stability in all microsatellite loci.

These data demonstrate that the presented microfluidic-based approach is a fast and reliable but also a very sensitive procedure for MSI analyses in clinical studies. Microfluidic-based technologies have the advantage not to depend on a label and thus not to require specialised procedures or equipment to detect the label (see supplementary table S2). In contrast, most of the conventional methods applied in MSI detection are dependent on fluorochrome-labelling of PCR amplicon, and these are subsequently analysed commonly by capillary electrophoresis using laser-associated sequencing platforms.

In conclusion, the microfluidic-based approach for MSI analyses is a highly time-efficient and easy procedure combined with high sensitivity for MSI detection.

**Take home message**

- Microfluidic-based on-chip electrophoresis enables very reliable and sensitive evaluation of microsatellite instability. Therefore, microfluidic-based chip electrophoresis is an adequate procedure suitable for research laboratories and fulfilling the Bethesda criteria for microsatellite instability detection while saving time and technical efforts.
